# Conversion of Waste Cooking Oil into Bio-Fuel via Pyrolysis Using Activated Carbon as a Catalyst

**DOI:** 10.3390/molecules28083590

**Published:** 2023-04-20

**Authors:** Warintorn Banchapattanasakda, Channarong Asavatesanupap, Malee Santikunaporn

**Affiliations:** 1 Department of Chemical Engineering, Faculty of Engineering, Thammasat University, Pathumthani 12120, Thailand; 2 Department of Mechanical Engineering, Faculty of Engineering, Thammasat University, Pathumthani 12120, Thailand

**Keywords:** waste cooking oil, pyrolysis, activated carbon, catalyst, biofuels, biomass

## Abstract

The utilization of activated carbon (AC) as a catalyst for a lab-scale pyrolysis process to convert waste cooking oil (WCO) into more valuable hydrocarbon fuels is described. The pyrolysis process was performed with WCO and AC in an oxygen-free batch reactor at room pressure. The effects of process temperature and activated carbon dosage (the AC to WCO ratio) on the yield and composition are discussed systematically. The direct pyrolysis experimental results showed that WCO pyrolyzed at 425 °C yielded 81.7 wt.% bio-oil. When AC was used as a catalyst, a temperature of 400 °C and 1:40 AC:WCO ratio were the optimum conditions for the maximum hydrocarbon bio-oil yield of 83.5 and diesel-like fuel of 45 wt.%, investigated by boiling point distribution. Compared to bio-diesel and diesel properties, bio-oil has a high calorific value (40.20 kJ/g) and a density of 899 kg/m^3^, which are within the bio-diesel standard range, thus demonstrating its potential use as a liquid bio-fuel after certain upgradation processes. The study revealed that the optimum AC dosage promoted the thermal cracking of WCO at a reduced process temperature with a higher yield and improved quality compared to noncatalytic bio-oil.

## 1. Introduction

The effective management of large amounts of waste cooking oil (WCO) generated from households and food industries is a crucial environmental issue. Selecting suitable technologies to handle the complexities of various WCOs is of paramount importance. Uncontrolled WCO dumping and landfilling without any treatment leads to environmental contamination and human health problems. Recently, WCO was used as a feedstock in bio-diesel production through transesterification [1,2]; however, the low cetane number and calorific value of bio-diesel are burdens for such utilization, while extraction costs in the glycerol-recovery process during transesterification are high [3]. In some countries, WCO management is achieved through incineration and combustion to mitigate the waste volume and support energy recovery. These processes recover only the calorific value and not the chemical value of the WCO. The combustion process also increases air pollution by generating carbon oxides, nitrogen oxides, sulfur oxides and fly ash.

New technologies to improve WCO recycling and treatment processes now focus on the recovery of WCO energy and chemical values. The pyrolysis technique is regarded as an effective, economical and environmentally friendly treatment process for many kinds of waste [4,5]. Applying the pyrolysis technique in the thermochemical processing of WCO offers a high potential to recover the energy and render valuable products (oil and gas) that can be used as feedstock in petroleum refineries and petrochemical industries.

WCO mainly consists of a mixture of triglycerides and fatty acids. Pyrolysis under the complete absence of oxygen yields more valuable products including oil, gases and char. The yield and quality of these pyrolytic products depend on the characteristics of the feedstocks, temperature, heating rate, type of reactor, residence time, types of catalyst and catalyst dosage as the basic parameters contributing to the efficiency of the WCO pyrolysis process. Pyrolysis has been widely used to transform triglyceride-based materials into energy such as vegetable oils (soybean oil, castor oil, palm oil) [6,7,8], glycerol [9], animal fats [10] and used cooking oil [11,12]. However, no studies have addressed the development of a practical simulated formula to compute or predict the yield and characteristics of the final pyrolytic products because of the complexity of the component chemical interaction and the influence of pyrolysis conditions [13].

Wiggers et al. [14] studied the continuous pyrolysis of soybean oil at temperatures ranging from 450 °C to 600 °C in a pilot plant and obtained bio-fuels similar to fossil fuels, while Lappi and Alén [15] examined thermochemical behaviors using gas chromatography and mass selective and flame ionization detection of bio-oil from saponified palm, olive, rapeseed and castor oil pyrolysis at 750 °C for 20 s. The results showed that bio-oils mainly contained linear alkenes (up to C19) and alkanes (up to C17), similar to those found in the gasoline (C4–C10) and diesel fuel (C11–C22) boiling range fractions of petroleum. More recently, Ben Hassen-Trabelsi et al. [16] investigated bio-fuel production from waste cooking oil via the pyrolysis process in a lab-scale fixed-bed reactor at temperatures between 550 °C and 800 °C and at different heating rates. The results showed that 800 °C and a heating rate of 15 °C/min was the optimum condition, yielding 80% bio-oil with a high calorific value (8843 kg/kcal). However, the high acidity and viscosity values were a serious limitation.

Catalysts are essential to improve the pyrolysis process for both bio-oil quality and quantity; zeolites are popular catalysts for pyrolysis. Buzetzki et al. [17] studied the influence of zeolite catalysts on pyrolysis products by thermal cracking of rapeseed oil in a batch reactor tested at 350–440 °C with a 1:10 catalyst to oil ratio. Liquid yields of 90–93 wt.% were obtained with a large diesel range and properties close to diesel fuel. Alumina and aluminosilicate-based catalysts have also been studied in triglyceride-based material pyrolysis. Most types of alumina catalyst generally encompass mesoporous materials such as MCM-41. Ngo et al. [6] pyrolyzed soybean oil with various catalysts, including H-ZSM5 and MCM-41, in a fixed-bed reactor at 420 °C and 450 °C under a nitrogen flow. The liquid products from the experiment using H-ZSM5 catalysts were mostly aromatic components, while those using metal/MCM41 catalysts were a mixture of alkanes, alkenes, alkadienes, aromatic and carboxylic acids.

Carbonaceous material such as activated carbon (AC) has been extensively studied as a catalyst in triglyceride pyrolysis, because of AC’s large surface area and high porosity, which are the most important properties of solid catalysts [18]. These properties of AC affect reactivity in the decomposition process, including pyrolysis, by allowing the efficient contact time between the pyrolytic feedstock and the active sites [19,20,21,22]. Therefore, AC helps the thermal cracking of pyrolytic feedstock into lighter molecules, giving higher amounts of gaseous products [19,22]. Not only the surface area and porosity properties but also the functional groups and acidic sites of AC greatly affect the AC reactivity in the pyrolysis process [19,21]. The acidic properties of AC are associated with the surface functional groups. The acid surfaces are presented by carboxylic, phenolic, lactone, carboxylic anhydride and peroxide groups [21], which depend on the type of feedstock material and the modified activation process in the AC production. The acidic character of AC makes the AC a hydrophilic substance, which is potentially expected to convert the feedstock into the aromatic hydrocarbons by the pyrolysis process [23,24,25]. Moreover, due to the carbon structure of AC, the benzene rings on AC’s surface can accelerate the cracking process of hydrocarbon molecules by increasing the length of C–C of hydrocarbons [19].

**Figure 1:**
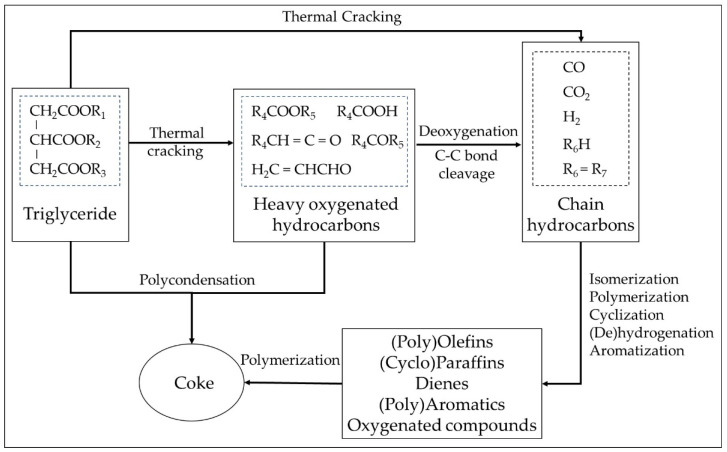
The reaction scheme for the pyrolysis of triglycerides over an activated carbon adapted from [20,26].

Since the pyrolysis process in WCO involves many highly complex organic reactions, there is no definite solution to the resulting reactions that will be obtained in each execution. Figure 1 presents the proposed reaction scheme for pyrolysis over an activated carbon [19,20,26]. Triglyceride is decomposed to long-chain heavy oxygenated hydrocarbons. The thermal-cracking reaction continues, together with deoxygenation reactions, forming lighter oxygenated hydrocarbons (carboxylic acids, alcohols, ketones, aldehydes, esters, etc.), chain hydrocarbons such as alkanes, alkenes and dienes and gaseous products (CO_2_, CO, H_2_ and light hydrocarbons). Then, many reactions, for example isomerization, polymerization, aromatization and cyclization, occur to form various kinds of products, such as saturated and unsaturated hydrocarbons, aromatics, cyclic hydrocarbons, light oxygenated hydrocarbons, etc.

Because of its heat tolerance properties and low cost, AC is also used for heating applications [27]. Heil [28] introduced the Greasoline process using an activated coal fixed-bed reactor to convert animal fat and vegetable oil into more valuable products at 450 °C to 600 °C. The results showed that the liquid product contained 55 wt.% diesel components. AC was also employed as an absorbent in the microwave pyrolysis of used frying oil [29,30,31]. The results showed that AC acted as both a medium (heat-transferring agent) and a catalyst for heating, and pyrolyzed WCO to yields of up to 73 wt.% bio-oil products. However, compared to the studies of WCO catalytic pyrolysis, there are very few reports of using AC as a catalyst. Most research has been to develop the WCO process and upgrade the bio-oil in WCO catalytic pyrolysis in using zeolites and aluminosilicates catalysts [3,6,11]. In addition, AC has been studied as just a heat-transferring agent in microwave-assisted pyrolysis studies [29,30].

Therefore, this research aimed to upgrade the WCO pyrolysis process in terms of bio-oil yield and quality characteristics using AC as the catalyst to enable effective waste management with energy saving as a critical environmental issue. The effects of the process temperature and AC dosage on product yields were investigated. The detailed characterizations of the bio-oil product at optimum conditions along with the proposed applications are also discussed.

## 2. Results and Discussion

### 2.1. The Characterization of WCO as Pyrolysis Feedstock and AC as a Catalyst

The characteristics of WCO were examined and are shown in Table 1. Carbon, oxygen and hydrogen were the major components of WCO at 78.69, 8.20 and 12.22 wt.%, respectively. The high calorific value of 39.1 MJ/kg suggested that WCO could be a suitable feedstock in the pyrolysis process, with the potential to recover the energy value from transforming WCO to a bio-fuel. Moreover, the very small amounts of nitrogen and no sulfur detected in WCO were considered to generate reduced pollutant emissions, such as NOx, SOx and NH_3_, during thermal cracking in the pyrolysis process. WCO primarily contained palmitic acid at 56 wt.%, oleic acid 33.72 wt.% and linoleic acid 7.53 wt.%. Thus, WCO predominantly consisted of saturated fatty acids. The results in Table 1 show that almost all the compounds (93.19 wt.%) in WCO were vaporized above 350 °C, relating to the boiling points of palmitic acid, oleic acid and linoleic acid [32]. Therefore, the minimum pyrolysis temperature of WCO was determined as 350 °C in this study.

**Figure 2:**
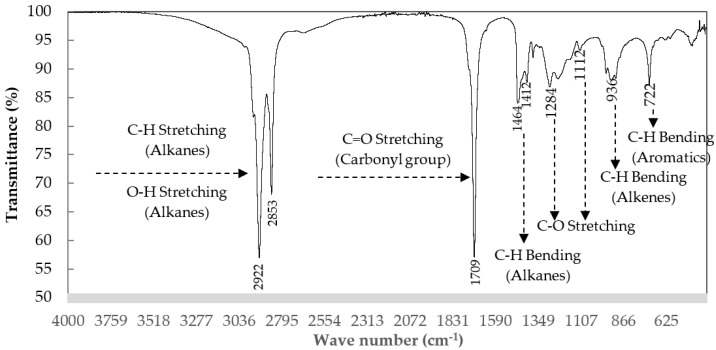
The FT–IR profile of the WCO used in this study.

The chemical structure of WCO examined by the FT–IR spectrum was also supportive, with results shown in Figure 2 and Table 2. A characteristic of WCO is its aliphatic hydrocarbons. A significant indicator of the aliphatic groups is the high intensity of the peak bands between 2921.91 cm^−1^ and 2852.83 cm^−1^ representing aliphatic C–H bonds such as alkanes and carboxylic acid O–H stretching. The weak intensity of the bands in the range 1464.30 cm^−1^ to 1412.34 cm^−1^ and peak bands at 935.95 and 721.89 cm^−1^ also represent aliphatic group characteristics of WCO. Oxygenated compounds were also detected at the peak band of 1708.93 cm^−1^ as C=O stretching and at 1283.64–1112.96 cm^−1^ as C–O stretching compounds.

**Figure 3:**
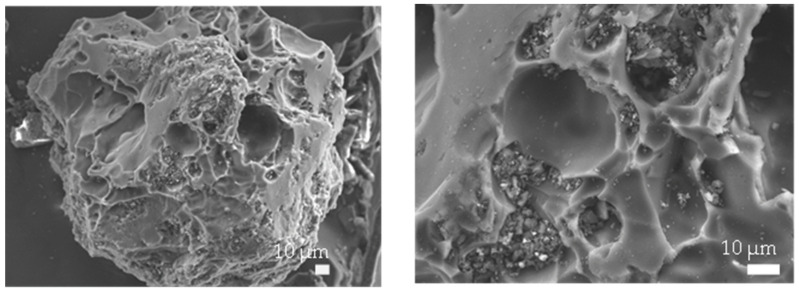
SEM images of activated carbon used in this study.

The physical and chemical properties of AC were characterized and shown in Table 3. The ultimate analysis data confirm that carbon is the dominant element of 72.33 wt.%. A BET surface area of 720.0 m^2^/g and average pore size of 0.95 nm were obtained from the adsorption/desorption of N_2_. The SEM images of AC morphology are shown in Figure 3. The morphology of AC is a granular shape with a high porous structure.

### 2.2. The Pyrolysis of WCO in the Absence of AC

**Figure 4:**
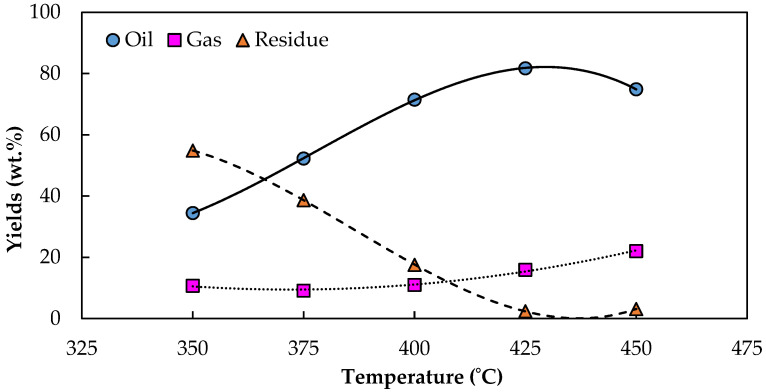
The influence of temperature on the product yields of pyrolysis reactions. (Operating conditions: a flow rate of N_2_ of 40 mL/L without a catalyst and a pyrolyzed temperature range of 350–450 °C).

Figure 4 illustrates the influence of the process temperature from 350 °C to 450 °C without a catalyst on the WCO pyrolysis yields of liquid, residue and gaseous products when using the same quantity of feedstock to ensure the maximum productivity of each yield under the same conditions. The results showed that the WCO samples were thermally cracked to pyrolysis products in different proportions at each process temperature. Initially, the pyrolysis performed at 350 °C demonstrated incomplete cracking, with half of the WCO remaining unpyrolyzed, and only a 35% bio-oil yield and 10% gaseous yield obtained. When the temperature was increased to 425 °C, the production of bio-oil increased to 81.70%, implying that the optimum pyrolysis temperature was at 425 °C. The maximum bio-oil yield achieved was 82%, with a minimum nonpyrolyzed (residue) yield of 2.3% and a 16% gaseous product yield. WCO pyrolysis is an endothermic process, described by the concentration of primary decomposition or depolymerization reactions to produce volatiles in bio-oil and gaseous forms [16]. At 450 °C, bio-oil yield dropped to 75%. The pyrolysis bio-oil products were decomposed by excess heat into gaseous products, as observed by the increased yield of the pyrolysis gaseous products from 16% to 22%. Sampath [33] also investigated the effect of temperature on the pyrolysis product yield of camelina seed oil at 300 °C to 600 °C; his results showed that liquid yield ranged from 45% to 75% of the total mass feed.

**Figure 5:**
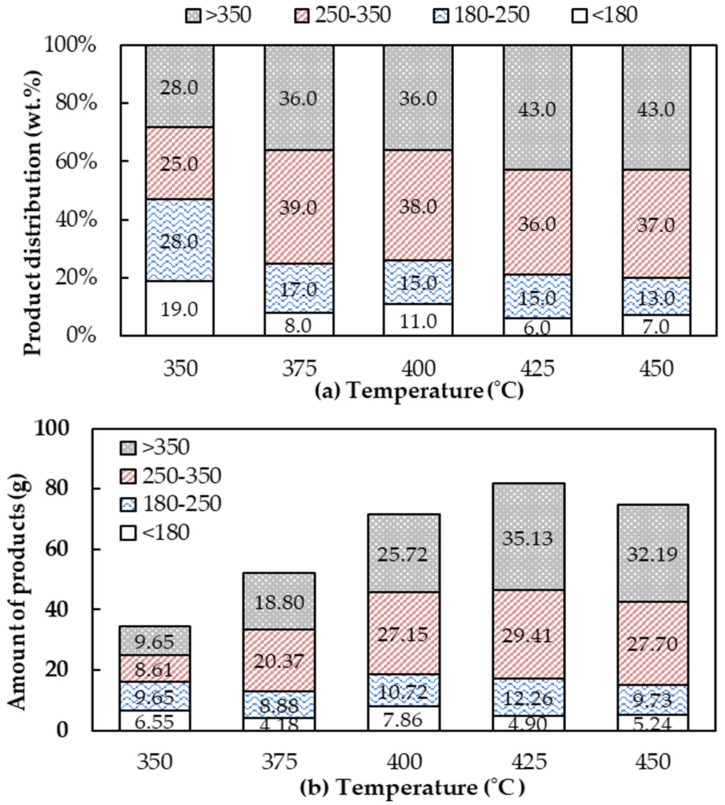
The boiling temperature distribution of liquid bio-oil obtained from pyrolysis. (**a**) Product distribution by percentage; (**b**) production distribution by amount. (Operating conditions: a flow rate of N_2_ of 40 mL/L without a catalyst and a pyrolyzed temperature range of 350–450 °C).

The distribution of bio-oil separated at different temperature ranges by GC–SIMDIS is presented in Figure 5a. At 425 °C, the optimum pyrolysis temperature, the major components in the bio-oil, were fuel oil-like compounds at 43% with boiling points higher than 350 °C. Then, the diesel-like compounds with a boiling temperature range of 250–350 °C (C15–C20) at 36.00% and kerosene-like compounds with boiling range of 180–250 °C (C11–C14) at 15.00%. Moreover, it can be observed that the boiling temperature distribution in the bio-oil obtained from 350 °C showed almost the same distribution in each boiling temperature range. However, when the temperature increased, the distribution of diesel-like compounds and fuel oil-like compounds increased up to 39% and 43%, respectively. It also proved that direct pyrolysis was the major decomposition stage of fatty acids in WCO and when the temperature increased, the production of hydrocarbons also increased, which promoted the pyrolysis of macromolecules [34]. To select the optimum condition for upgrading to a more valuable alternative energy, the product yield as shown in Figure 5b and other properties must be considered. A comparative analysis of the economic aspects of these outcomes should also be conducted.

### 2.3. The Pyrolysis of WCO in the Presence of AC

#### 2.3.1. The Effect of Reaction Temperature and AC

**Figure 6:**
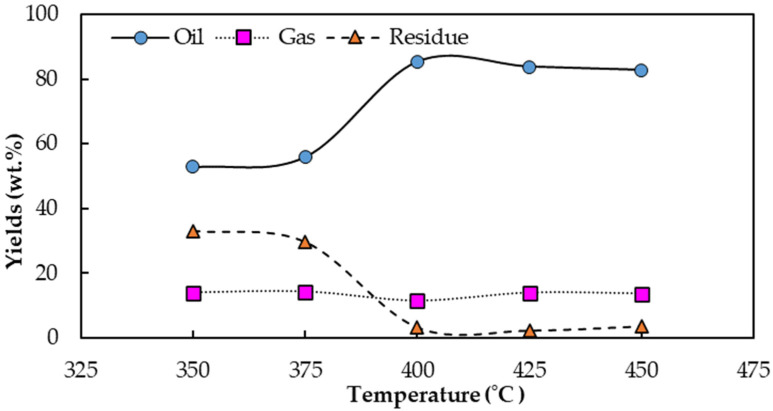
The influence of reaction temperature on product yields. (Operating conditions: a flow rate of N_2_ of 40 mL/L, an AC:WCO mass ratio of 1:40 and a pyrolyzed temperature range of 350–450 °C).

The influence of reaction temperature on the product yield was assessed at 350, 375, 400, 425 and 450 °C by fixing the ratio of AC to WCO at 1:40 to investigate the optimum temperature in terms of the maximum bio-oil yield. As shown in Figure 6, the bio-oil yield increased with temperature of 350 °C to 400 °C, with maximum yield at 400 °C of 85.35%, and then declined at higher temperatures. This phenomenon was described in the previous section. Higher reaction temperatures increased the thermal cracking reactions in the pyrolysis process by breaking the carbon chain compounds and contributing to the volatilization of WCO, called the primary decomposition [16]. The secondary decomposition occurred when the temperature was increased to 425 °C or higher, causing a reduced production of bio-oil at 83.85%, with 82.81% at 450 °C.

These results followed the same trend as the product yield of WCO pyrolysis without AC in the previous section, but differed in some interesting aspects. The bio-oil yield at all reaction temperatures in the presence of AC pyrolysis increased, especially at 350 °C from 34.45% to 52.85%, together with a reduction in the residue yield from 54.85% to 33.13%. Moreover, the optimum endothermic reaction temperature in terms of the maximum bio-oil production shifted from 425 °C to 400 °C, with a similar increase in bio-oil yield from 81.70% to 85.35%. The AC acted as a catalyst and promoted the reaction temperature in the pyrolysis process beyond 425 °C due to the electrostatic forces [19]. Additionally, the catalytic effect on WCO occurred on the entire high surface area of the AC in its pores, so helped the thermal decomposition of triglycerides in WCO into lighter molecules and produced a large amount of vapor products [23], while the secondary decomposition reduced. Interestingly, reducing the optimum pyrolysis temperature using a small amount of AC resulted in energy saving with the efficiency maintained.

#### 2.3.2. The Effect of the AC:WCO Ratio

The influence of AC:WCO ratios at 1:40, 1:30 and 1:20 on the pyrolysis process under two reaction temperatures of 350 °C and 400 °C was also examined, as shown in Figure 5. The yield of bio-oil, residue and gaseous products altered, especially for bio-oil and residue.

**Figure 7:**
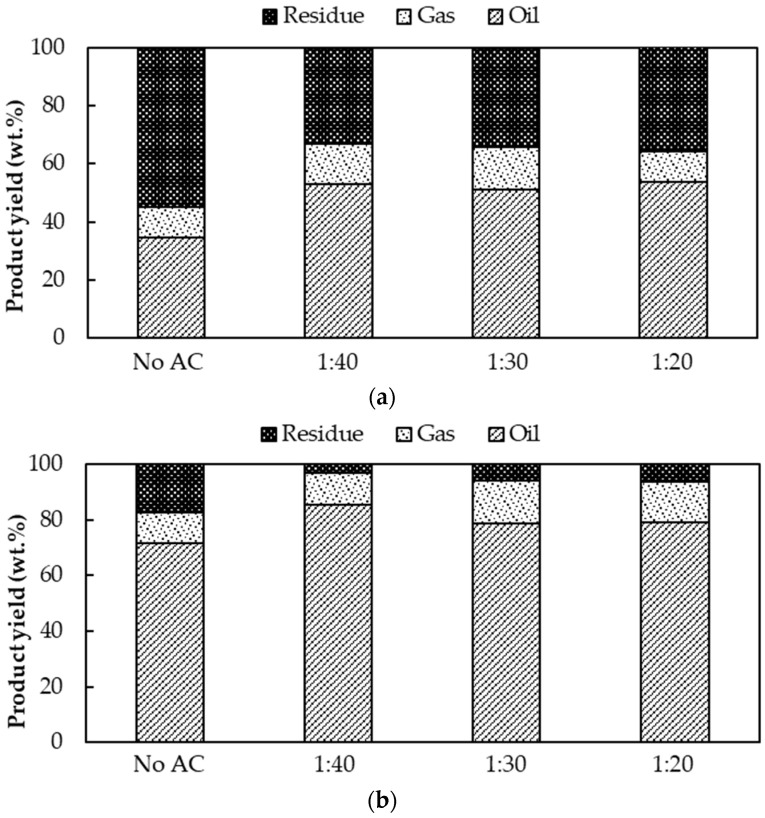
The product yield distribution obtained from pyrolysis at various AC:WCO mass ratios at the process temperature (**a**) at 350 °C; (**b**) 400 °C.

At 350 °C (Figure 7a), the change in the AC:WCO ratio did not significantly affect the pyrolysis product yields, whereas at 400 °C (Figure 7b), the change in the AC:WCO ratio significantly impacted the pyrolysis product yields. At an AC:WCO ratio of 1:40, the maximum bio-oil yield was achieved at 85.35%, with a minimum nonpyrolyzed (residue) yield of 3.16% and 11.49% gaseous product yield, similar to the results of direct pyrolysis (an absence of AC) at 425 °C. This occurred because the AC catalyst at a 1:40 AC:WCO ratio enhanced the process temperature from 400 °C to the final temperature at almost 425 °C. With a small increase in the of AC catalyst, the yield of bio-oil gradually decreased from 85.35% to 77.72% and 73.32%. However, the yield of the residue did not significantly change at 3.16%, 5.91% and 5.65%, respectively. The bio-oil yield significantly reduced when the AC amount increased (1:30 and 1:20) because, with the higher the amount of AC used, there was a higher surface contacting area in the WCO pyrolysis process. This reactivity of AC enhanced the process temperature beyond 425 °C as the optimum pyrolysis temperature. The AC also caused the thermal-cracking secondary decomposition of WCO, resulting in a higher conversion of some WCO to pyrolysis gases [35]. This further decomposed the pyrolysis bio-oil product into gaseous products, as described in the previous section.

**Figure 8:**
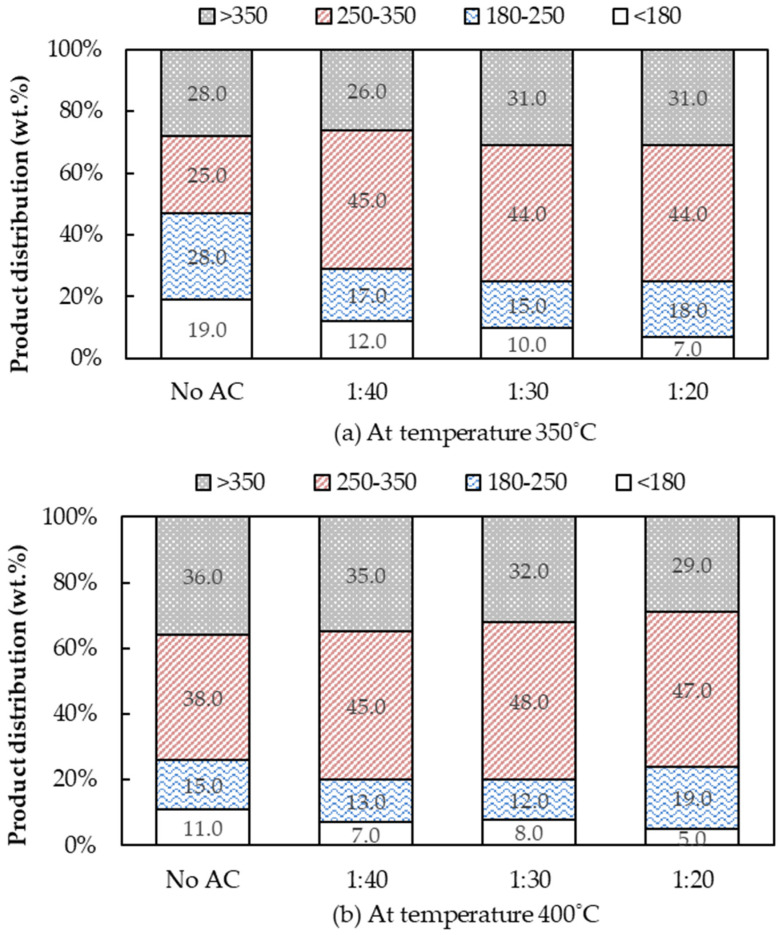
The boiling temperature distribution of the liquid bio-oil obtained from pyrolysis of WCO at various AC:WCO mass ratios. (Operating conditions: a flow rate of N_2_ of 40 mL/L; a pyrolyzed temperature at 350 and 400 °C).

As shown in Figure 8a,b, the boiling temperature distribution obviously changed when the AC was used as a catalyst, especially for the bio-oil obtained from the process temperature at 350 °C. The amount of bio-oil in the boiling range of fuel oil-like fuel (>350 °C; C > 20) did not greatly alter with AC usage. Conversely, significant changes occurred in the amount in the boiling point range of gasoline-like fuel, kerosene-like fuel and diesel-like fuel. The proportion of diesel-like fuel significantly increased compared to those of non-AC usage, from 25 wt.% to 45 wt.% for bio-oil obtained from the process temperature at 350 °C and from 38 wt.% to 48 wt.% for the bio-oil obtained from the process temperature at 400 °C. Interestingly, the amount in each boiling point range did not significantly change when the AC dosage increased at a low reaction temperature. At the optimum conditions for the maximum bio-oil yield, a WCO:AC ratio of 1:40 at 400 °C, 45 wt.% of bio-oil was in the boiling point range of diesel-like fuel (250–350 °C), 35 wt.% fuel oil-like fuel (>350 °C), 13 wt.% kerosene-like fuel (180–250 °C) and 7 wt.% gasoline-like fuel (<180 °C). It was revealed that the AC as a catalyst enhanced the WCO pyrolysis to produce more valuable products such as diesel-like compounds in the liquid bio-oil. As mentioned before, the AC acted as a heat-transfer agent that enhanced the reaction temperature in the pyrolysis process, promoting thermal cracking from long carbon-chain compounds to form low carbon-chain compounds in the bio-oil. Therefore, when selecting the optimum condition to upgrade to a more valuable alternative energy, the product yield and other properties must also be considered to secure the most suitable economic value.

### 2.4. The Bio-Oil Characteristics

The bio-oil produced from WCO pyrolysis with an AC:WCO ratio of 1:40 at 400 °C was analyzed based on the chemical compositions determined by GC–MS. In this study, pyrolysis gases and char residue were excluded from the analysis due to the low yields obtained. The detected qualitative organic compounds were listed according to increased retention times and their areas were determined and summarized in Table 4.

The bio-oil consisted of a complex mixture of 27.03 wt.% hydrocarbon compounds and 72.97 wt.% oxygenated compounds, as shown in Table 4. The compounds containing carbon atoms ranged from C10 to C27 with trace amounts of C36. The oxygenated compounds in bio-oil were 52.33 wt.% carboxylic acids, 11.12 wt.% alcohols, 3.67 wt.% ketones, 2.86 wt.% aldehydes and 2.30 wt.% esters. The hydrocarbons found in bio-oil were 17.48 wt.% alkanes, 6.53 wt.% alkenes and 3.02 wt.% alkynes. Many studies reported on the similarities of GC–MS compositions of bio-oils produced from the pyrolysis of various triglyceride materials. Ben Hassen-Trabelsi et al. [16] investigated the GC–MS chemical compositions of a pyrolytic oil produced from waste cooking oil in a pyrolysis process using a fixed-bed reactor. The results showed that the bio-oil contained organic compounds with carbon atoms ranging from C6 to C27 as the major components, explaining the predominant presence of 89.61 wt.% carboxylic acid, 2.31 wt.% linear saturated hydrocarbons, 3.64 wt.% linear unsaturated hydrocarbons, 0.17 wt.% cyclic hydrocarbons, 1.86 wt.% alcohols, 0.25 wt.% ketones and 0.25 wt.% aldehydes (by calculation from the GC–MS result reported [16]). Similarly, Kraiem et al. [36] reported that GC–MS compositions of bio-oil obtained from the pyrolysis of waste frying oil consisted of oxygenated compounds such as 53.11 wt.% carboxylic acids, 1.71 wt.% aldehydes, 1.21 wt.% ketones, and 0.93 wt.% alcohols, 4.03 wt.% linear saturated, 28.65 wt.% unsaturated and 9.54 wt.% cyclic hydrocarbons (by calculation from the GC–MS result reported [36]). These studies demonstrated the significant hydrocarbon compositions of bio-oils as carboxylic acids related to high fatty-acid compositions in WCO.

**Figure 9:**
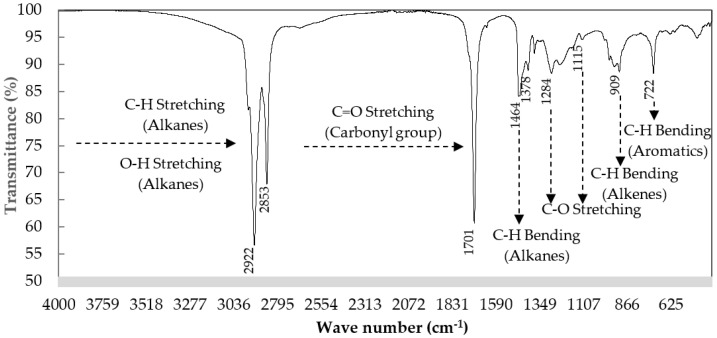
The FT–IR spectra of liquid bio-oil obtained at the optimum operating conditions.

Figure 9 presents the FT–IR spectra of liquid bio-oil obtained at the optimum operating condition. The intense peaks between 2921.81 and 2852.74 cm^−1^ represent carboxylic acids (O–H stretching) together with alkanes (C–H stretching). The high intensity of these bands highlights the dominance of saturated aliphatic hydrocarbons in bio-oil. The presence of other aliphatic groups (C–H bending) was shown by bands between 1464.30 and 1377.59 cm^−1^, confirming the aliphatic character of bio-oil. The presence of oxygen in bio-oil was represented by the signal of C=O stretching at 1709.26 cm^−1^, indicating carbonyl groups such as aldehydes, ketones, esters and carboxylic acids. The C–O stretching vibration was affirmed by bands in the range 1284.02 to 1115.14 cm^−1^ representing esters, ethers, alcohols and carboxylic acids. The weak bands between 909.66 and 721.75 were probably due to C–H bending, such as alkenes and aromatic hydrocarbons in the bio-oil. The bio-oil obtained from this study was influenced by carboxylic acids, aliphatic hydrocarbons and small amounts of aromatics, aldehydes and ketones, related to the group of organic compounds investigated by GC–MS analysis described in Table 4.

In comparison to the study of the FT–IR spectra of diesel fuel and bio-diesel produced from the mixture of transesterified waste canola and waste transformer oils by Qasim et al. [37], it was found that the FT–IR spectra of liquid bio-oil was similar to diesel and bio-diesel fuel in terms of high intensity of aliphatic compounds. However, there was a significant difference in the way that the liquid bio-oil had intensity peaks of oxygenated compounds. These could indicate that the WCO catalytic co-pyrolysis in this study could produce a value-added liquid fuel that could potentially be upgraded to commercial diesel fuel.

### 2.5. The Fuel Properties of Liquid Bio-Oil

To complement the chemical compositions and functional groups of bio-oil, physical properties including the density, kinematic viscosity, water content, calorific value and acid value of bio-oil obtained from catalytic pyrolysis at an AC:WCO ratio of 1:40 and 400 °C were also analyzed and compared with diesel and bio-diesel obtained from the transesterification process, as shown in Table 5.

Table 5 shows the calorific value of bio-oil at 40.20 kJ/g, lower than the diesel petroleum fuel standard (>45 kJ/g) and bio-diesel (>42 kJ/g), but higher than the original WCO before pyrolysis (39.07 kJ/g) and noncatalytic liquid bio-oil (39.73 kJ/g). This occurred because of the similar high carbon and hydrogen bio-oil contents and also the low oxygen content. In previous calorific value studies of bio-oils obtained from triglyceride materials, Lam et al. [27] reported calorific value at 40–46 kJ/g for bio-oils obtained from the microwave pyrolysis of frying oil, while Ben Hassen-Trabelsi et al. [16] reported a heating value of bio-oil at 36.9 kJ/g, lower than the diesel and bio-diesel standards. The high calorific value of bio-oil from WCO pyrolysis with AC as a catalyst showed a high potential for use as an engine fuel or as chemical feedstock to produce synthetic fuels.

The water content of bio-oil was 0.23%, conforming to the standards for diesel and bio-diesel of less than 0.5%. The amount of water in the bio-oil is important because even fine droplets of water can cause damage in an engine’s combusting cylinders and block filters. Moreover, a high water content also reduces the calorific value of bio-oil.

The experimental catalytic bio-oil had a density at 15 °C of 899 kg/m^3^, higher than the density range of diesel petroleum fuel standards (820–845 kg/m^3^), but within the range of bio-diesel density at 820 to 900 kg/m^3^. This result concurred with previous works [13,16,38] that recorded a density of pyrolytic bio-oil produced from waste cooking oil at 899 kg/m^3^, indicating that bio-oil can be used directly as engine fuel. Wan Mahari et al. [39] reported a low density of liquid fuel obtained from used cooking oil by microwave co-pyrolysis, ranging from 764 to 790 kg/m^3^.

The kinematic viscosity value of that bio-oil at 40 °C was 11.11 cSt and higher than for bio-diesel (1.8–4.5 cSt) and 1.8–4.1 cSt for diesel fuel, but in the same range as previous studies [40] of pyrolytic bio-oils from triglyceride materials.

The acid values of noncatalytic bio-oil and catalytic bio-oil in this study were 118.61 and 126.78 mgKOH/g, respectively, which are similar to 126.8 mgKOH/g [16], 124.34 mgKOH/g [10] and 124.56 mgKOH/g [36] in other research. This strong acidity indicated the presence of high contents of free fatty acids, especially carboxylic acid, and related to the results of FT–IR and GC–MS analyses. The thermos-cracking process produces acids from triglycerides in WCO [11]. The acidic components in bio-oil must be reduced for use as engine fuel because high acid components cause corrosion in fuel storage and also in other components of the engine system. One technique to reduce acidity and also enhance the properties of bio-oil is catalytic esterification [41]. As seen from previous work, the acid value of bio-diesel obtained from the transesterification of waste cooking oil was 0.4 mgKOH/g [42].

The hydrogen to carbon ratio (H/C) is a basic characterization indicator of the presence of hydrocarbon fuels and pyrolysis oils. Alterations in the H/C molar ratio indicate changes in C–C bond saturation [43]. The higher the H/C ratio, the higher the energy performance of the fuels. In this study, an increase in atomic H/C ratio from 1.85 to 1.88 was observed for catalytic bio-oils compared to WCO, within the ranges of bio-diesel and diesel specifications. Dehydrogenation and aromatization occurred in the WCO pyrolysis process, leading to the formation of C=C bonds, such as alkenes and aromatic compounds that were recorded in GC–MS and FT–IR analyses. Hydrogenation is one of the techniques that should be conducted to increase the H/C ratio of catalytic liquid bio-fuels.

The elemental composition of catalytic liquid bio-oil compared to WCO, noncatalytic bio-oil, bio-diesel and diesel is shown in Table 5. From the analysis, it can be observed that the carbon and hydrogen contents of catalytic bio-oil are 79.64 wt.% and 12.59 wt.%, respectively, which are slightly higher than those of WCO, noncatalytic bio-oil and bio-diesel, but lower than those of diesel. It can be said that catalytic bio-oil has superior contents of carbon and hydrogen that possibly deliver more power to the engine than bio-diesel but, of course, less than the diesel fuel as shown by the calorific values in Table 5. An important element difference between catalytic bio-oil and diesel fuel is the relatively high content of oxygen (7.4%) in catalytic bio-oil. However, its oxygen content is less than that of biodiesel (8.90–11.15%). The oxygen composition in bio-oil is from the oxygenated compounds, including carboxylic acids, alcohols, ketones, aldehydes and ester, as described in the previous section. Moreover, it could be said that AC as a catalyst in WCO pyrolysis could reduce the oxygen content compared to the oxygen content of noncatalytic bio-oil. Interestingly, the pyrolytic bio-oil has a low amount of nitrogen (0.37–0.47 wt.%) and no sulfur detected, which results in lower emissions of NO_x_, SO_x_ and NH_3_ to the atmosphere.

**Table 5:** The fuel properties of WCO, liquid bio-oil, diesel and bio-diesel.

Fuel Properties	WCO	Noncatalytic Bio-Oil ^a^	Catalytic Bio-Oil ^a^	Bio-Diesel ^b^	Diesel ^c^
Elemental analysis, wet basis					
- Carbon (C)	78.69	77.19	79.64	76.66–79.50	85.72–86.60
- Hydrogen (H)	12.22	12.18	12.59	10.30	13.2–13.4
- Nitrogen (N)	0.89	0.47	0.37	1.30	<0.2
- Oxygen (O) ^d^	0.2	10.16	7.40	8.90–11.15	-
- Sulfur (S)	-	-	-	-	<0.3
H/C ratio	1.85	1.88	1.90	1.86–1.88	1.77–1.95
Density at 15 °C (kg/m^3^)	910.10	877	899	820–900	810–870
Viscosity at 40 °C (cSt.)	42.50	8.03	11.16	1.8–4.5	1.8–4.1
Water content (%)	0.465	0.23	0.23	<0.5	<0.5
Calorific value (kJ/g)	39.07	39.73	40.20	>42.00	>45.00
Acid value (mgKOH/g)	5.4	128.61	126.78	0.2	5.0

^a^ Bio-oil obtained from this study; ^b^ specification of bio-diesel [44,45,46,47,48]; ^c^ specification of diesel [45,46,47,48,49]; ^d^ from mass balance.

### 2.6. Energy Recovery

**Figure 10:**
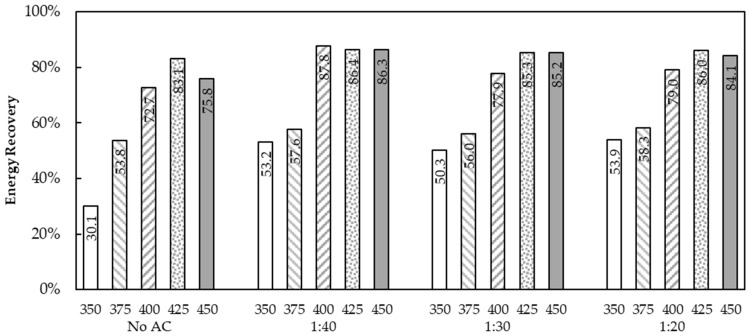
The energy recovery of the liquid bio-oil obtained from WCO pyrolysis.

The energy recovery of the liquid bio-oil obtained from the WCO pyrolysis at various temperatures and AC dosages calculated by Equation (1) to find the optimum conditions based on the calorific value of WCO and liquid bio-oil together with the yield of liquid bio-oil is shown in Figure 10. It was found that the liquid bio-oil from the process temperature of 400 °C at the AC dosage of 1:40 showed the highest energy recovery by 87.8% followed by bio-oil from the process temperature of 425 °C and 450 °C at the same AC dosage, respectively. It could be concluded that the WCO pyrolysis at 400 °C using AC as a catalyst at dosage 1:40 was the optimum condition in this batch operation study.

## 3. Materials and Methods

### 3.1. Materials

Dark-yellowish WCO, collected from leftover frying palm oils, was supplied by Thanachock Vegetable Oil Ltd., Samut Sakhon, Thailand, and used as a feed for pyrolysis without any pretreatment. Activated carbon (Biocat activated carbon CS1100; AC) with a particle size of 0.5–1.0 mm was used as a catalyst.

### 3.2. Catalytic Testing

**Figure 11:**
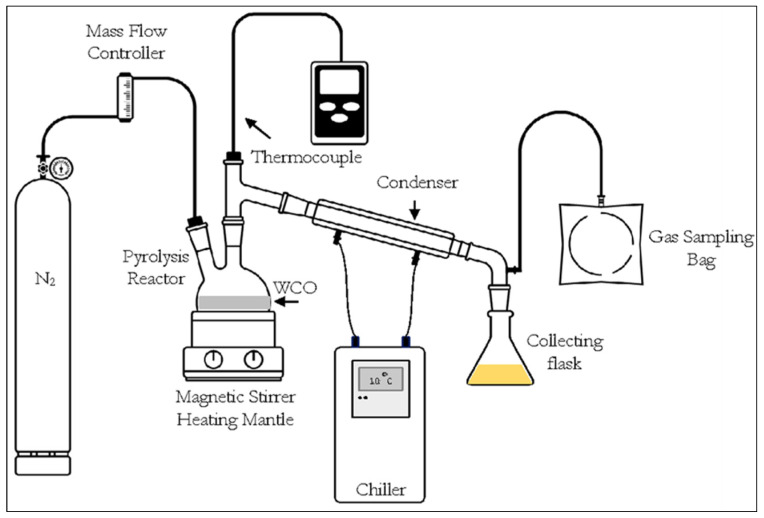
The schematic diagram of the experimental setup.

Pyrolysis experiments were conducted as a laboratory-scale batch operation, as shown in Figure 11. In each batch operation, 100 g of WCO was mixed with AC at mass ratios of 1:40, 1:30 on 1:20 and placed in a 1 L two-necked quartz reactor. Before use, the AC was heated at 110 °C for 12 h to remove moisture.

The pyrolysis system was validated with no leakages, and nitrogen was purged through the system with a flow rate of 40 mL/min to remove the oxygen from the system and ensure pyrolysis under oxygen-free conditions. The system was purged with nitrogen for at least 10 min before heating. A stainless-steel thermocouple probe was immersed inside the reactor in direct contact with the WCO sample. The reaction temperature was monitored throughout the pyrolysis experiments while stirring at 40 rpm, and heating by an electric magnetic stirrer heating-mantle until reaching the targeted pyrolysis temperatures ranging between 350 °C and 450 °C, with monitoring for 30 min.

In the experiment, the gaseous product generated during the pyrolysis process passed to the condenser operated at 10 °C with a circulating chilled water. The liquid product (bio-oil) was condensed and collected in a flask, while the noncondensable gases were collected in a gas sampling bag for further studies. The nonpyrolyzed residues were collected from the bottom of the reactor after cooling at room temperature. The pyrolysis experiments were conducted twice under the same reaction conditions and the mean value of product yields was calculated. The yields of the liquid and residue products were measured by weight, and by mass balance for gaseous products. Moreover, the energy recovery of the liquid bio-oil obtained from the WCO pyrolysis was calculated by the formula, as shown in Equation (1).

(1) $$Energy\;recovery,\%=\frac{\left(Weight\;of\;liquid\;bio-oil)(liquid\;bio-oil’s\;calorific\;value\right)}{\left(Weight\;of\;WCO)(WCO’s\;calorific\;value\right)}\times 100$$

### 3.3. AC and WCO Characterization and Product Analysis

Scanning electron microscope (SEM) was carried out on a JEOL JSM-IT300, Oxford X-Max 20 (Boston, MA, USA) to study the morphology of AC. An elemental analyzer (CHN628/O628, LECO Instruments, was used to determine the amount of carbon, hydrogen and nitrogen in the WCO and AC samples. The fatty acid compositions of WCO and the chemical compositions of bio-oil products were analyzed using a 7890B/5977A-MSD-GC-MS HP (Agilent, Santa Clara, CA, USA) equipped with a mass spectrometer detector. An HP-5MS capillary column (length 30 m, 0.25 mm, ID, 0.25 µm df.) was used to separate the constituents. The bio-oil products were diluted in acetone to reduce the viscosity and concentration, then a 0.2 µL sample solution was injected into the column at 250 °C with a split ratio of 10:1. Helium was applied as the carrier gas with a flow rate of 1 mL/min. The oven temperature was programmed from 100 °C to 300 °C at a heating rate of 10 °C/min, held at the initial temperature for 2 min and the final temperature for 20 min. The detector and ion source temperatures were 300 °C and 230 °C, respectively. The peaks were observed, and the compounds were identified from the W10N14 library. Fourier transform-infrared (FT–IR) spectroscopy together with a Nicolet iS50, (Thermo Scientific, Waltham, MA, USA) was used to determine the functional groups of WCO and bio-oil products, with spectra conducted at wavelengths between 4000 and 400 cm^−1^. Thermal analyses of the samples were determined by thermogravimetric analysis (TGA2, Mettler Toledo, Greifensee, Switzerland) by heating from 30 °C to a final temperature of 650 °C at a heating rate of 10 °C/min in a flow of nitrogen. This was to characterize the boiling point distribution of the hydrocarbon mixtures of bio-oil up to a boiling point of 1013 °F (545 °C) in compliance with ASTM D2887 or up to 963 °C (517 °C) according to ASTM D86. Different boiling temperatures related to the number of carbon atoms found in bio-oil were analyzed by a gas chromatograph, equipped with simulated distillation (Hewlett-Packard, HP6890 Agilent, Santa Clara, CA, USA). The boiling point of the hydrocarbon C4–C10 (gasoline) is below 180 °C, C11–C14 (kerosene) compounds have a range of 180–250 °C, C15–C20 (diesel) compounds have a range of 250 °C to 350 °C and carbon atoms above C20 (fuel oil) are distilled at temperatures above 350 °C [28].

To determine the fuel properties, the bio-oil samples were tested following the ASTM method. The density was measured on a K86200 automatic digital densitometer with a ASTM-D1250 standard. To determine the kinematic viscosity of bio-oil at 40 °C, the ASTM-D445 method was applied on a Tanaka KV-5S viscometer. The calorific values of the bio-oil samples were determined based on an ASTM D240 using an IKA C600 Global Standard Bomb Calorimeter. The acid value of the bio-oils was measured by the titration method following the ASTM-D664, while a hydrometer was used to determine the density at 15 °C, and the water content was determined using a Mettler Toledo Coulometric Karl Fischer Titrator.

## 4. Conclusions

The thermo-cracking technique or pyrolysis process can be used to convert waste cooking oil into useful energy. The results revealed that temperature was the significant parameter that affects the quantity of the bio-oil yield. Activated carbon in a small dosage (a 1:40 AC to WCO ratio) showed good catalytic performance and enhanced the pyrolysis process by reducing the optimum batch process temperature, with the maximum bio-oil obtained (85.35%) at a lower temperature (400 °C) compared to direct pyrolysis (425 °C) with no activated carbon as a catalyst (81.7%). Under the optimum conditions, the bio-oil had a high proportion of high calorific value diesel-like fuel with a low oxygen content and no sulfur. The bio-oil also met the density value standard of bio-diesel. However, the high acid value (126.78 mgKOH/g) and high viscosity of bio-oil (11.16 St) are major problems that require further study.

## Figures and Tables

**Table 1:** The characteristics of WCO.

Elemental Composition	(wt.%)
- C	78.69
- H	12.22
- N	0.89
- S	0.00
- ^a^ O	8.20
**Fatty Acid Composition**	**(wt.%)**
- Palmitic Acid (C_16_H_32_O_2_)	56.00
- Oleic Acid (C_18_H_34_O_2_)	33.72
- Linoleic Acid (C_18_H_32_O_2_)	7.53
- Others	2.75
^b^ **Distribution of Compounds**	**(wt.%)**
- <180 °C	0.13
- 180–250 °C	0.80
- 250–350 °C	5.66
- >350 °C	93.19
Calorific Value (MJ/kg)	39.10
Acid Value (mg KOH/g)	5.40

^a^ By a mass balance; ^b^ by thermogravimetric analysis.

**Table 2:** The FT–IR functional group of WCO.

Wave Number (cm^−1^)	Functional Group
2921.91–2852.83	C–H stretching (alkanes) Carboxylic acid O–H stretching
1798.93	C=O stretching (carbonyl group such as aldehydes, ketones, esters, anhydrides, carboxylic acids)
1464.30–1412.34	C–H bending (alkanes)
1283.64–1112.96	C–O stretching (ester, ethers, alcohols, carboxylic acids)
935.95	C–H bending (alkene)
721.89	C–H bending (aromatics)

**Table 3:** The characteristics of activated carbon.

Properties	
Ultimate analysis	Amount (wt.%)
- C	72.33
- H	1.97
- N	1.04
- * O	24.66
BET surface area	720.0 m^2^/g
Total pore volume	0.41 cm^3^/g
Average pore size	0.95 nm

* By a mass balance.

**Table 4:** The group of organic compounds investigated by the GC–MS analysis of liquid bio-oil.

Compounds	wt.%
Without oxygen atoms	
- Alkanes	17.48
- Alkenes	6.53
- Alkynes	3.02
With oxygen atoms	
- Carboxylic acids	52.33
- Alcohols	11.12
- Esters	2.30
- Ketones	3.67
- Aldehydes	2.86
- Others	0.69

## Data Availability

Not applicable.

## Sample Availability

Samples of the compounds are available from the authors.
